# Cultural adaptation of a scalable psychological intervention for Burundian refugee adolescents in Tanzania: a qualitative study

**DOI:** 10.1186/s13031-021-00391-4

**Published:** 2021-09-27

**Authors:** Aneeha Singh, Ashley Nemiro, Aiysha Malik, Marie-France Guimond, Estella Nduwimana, Samuel Likindikoki, Jeannie Annan, Wietse A. Tol

**Affiliations:** 1 International Rescue Committee, 122 E 42nd Street, New York, NY 1068 USA; 2 Independent Consultant, London, UK; 3 International Organization for Migration, Kabondo Ouest, Av. Ririkumutima, 13, Bujumbura, Burundi; 4 Muhimbili University of Health and Allied Sciences, United Nations Road, Dar es Salaam, Tanzania; 5 Section of Global Health, Department of Public Health, CSS, Øster Farimagsgade 5, bg 9, DK-1014 Copenhagen K, Denmark; 6 HealthRight International, 14 E 4th Street, New York, NY 10012 USA; 7 Department of Mental Health, Bloomberg School of Public Health, Johns Hopkins University, 624 N. Broadway, 8th Floor, Baltimore, MD 21205 USA

**Keywords:** Adaptation, Psychological intervention, Adolescents, Psychological distress, Refugees

## Abstract

**Background:**

There is an urgent need for evidence-based, scalable, psychological interventions to improve the mental health of adolescents affected by adversity in low-resource settings. Early Adolescents Skills for Emotions (EASE) was developed by the WHO as a brief, transdiagnostic, group intervention for early adolescents exhibiting internalising problems, delivered by trained and supervised lay providers. This study describes the cultural adaptation of EASE for Burundian adolescents living in Mtendeli refugee camps in Tanzania.

**Methods:**

A phased approach to adaptation of the EASE intervention and its implementation, was adopted and comprised of: (1) a desk review to synthesize existing research on mental health issues in conflict-affected Burundian communities, (2) a rapid qualitative assessment involving free listing and key informant interviews with multiple stakeholders, (3) cognitive interviews with end users, and (4) a two-part adaptation workshop involving the implementing partner staff, members of the refugee community and mental health experts. We applied the Bernal framework to systematically document and track adaptations across eight dimensions of the intervention.

**Results:**

Problems associated with worry, stress, sadness, shame and fear were identified as amongst the most critical mental health concerns, alongside a range of experiences of different forms of violence (such as gender-based violence, violence when fleeing from their homes) and associated problems. Problems associated with violence that included past experiences of fleeing as well as ongoing problems of gender-based violence in the camp. The most significant adaptations that were required included providing options for low literacy of participants, safety planning to address the high prevalence of sexual violence, simplification of strategies for the benefit of the end users and of lay facilitators, and implementation changes to consider involvement of refugee incentive workers. A majority of changes were across dimensions of language, people, metaphors, content, methods and context, while there were fewer changes regarding the goals and concepts of EASE.

**Conclusions:**

The approach to adaptation of a psychological intervention suggested both minor and major required changes. Adaptations based on the findings of this study are anticipated to enhance relevance and acceptability of the EASE intervention and its delivery for camp-residing Burundian refugees in Tanzania.

**Supplementary Information:**

The online version contains supplementary material available at 10.1186/s13031-021-00391-4.

## Background

Currently, the world is experiencing the highest recorded numbers of refugees since World War II with 1 % of the world’s population displaced [33]. The majority (85%) of the world’s 26 million refugees are hosted in low- and middle-income countries (LMIC) and 40% of the global refugee population are children under the age of 18 years [33]. The multiple risks to development and mental health faced by young people and their families during conflict are further compounded through displacement in LMIC contexts [30], particularly camp settings, where risks include inconsistent access to nutrition, interruption to education, poor sanitation, limited access to health care and child protection risks including sexual assault and violence [11, 24].

Low-cost and effective solutions are required to overcome the financial, workforce and infrastructural barriers to meeting the mental health needs of displaced young people and their families in LMICs. Evidence-based, scalable, psychological interventions demonstrate promise for feasible implementation in LMIC settings due to their briefness, simplicity and ability to be delivered by trained and supervised lay providers [26, 42]. However, there is limited knowledge on interventions that may be effective in improving the mental health of children and adolescents in LMICs. A Cochrane systematic review and meta-analysis of mental health treatments in low-resource humanitarian settings found limited evidence on psychological treatments that reduced posttraumatic stress disorder (PTSD) symptoms in children and adolescents (3 randomized controlled trials) [22]. A review of conflict-affected children in low-resource settings concluded that interventions overall appeared to show moderate effects, but the overall quality of evidence was low and often limited to specific subgroups and outcomes [19].

Against the background of limited evidence for psychological interventions for adversity-affected children and adolescents, further rigorous research is required. In response to this need, the World Health Organization (WHO) has embarked on a program of work to develop and test the effectiveness of potentially scalable psychological interventions as part of a broader initiative to strengthen coverage and quality of mental health services globally [40]. For example, individual- and group- delivered Problem Management Plus (PM+), has demonstrated feasibility and effectiveness for symptoms of psychological distress for women affected by gender-based violence living in informal settlements in Kenya [5] and conflict-affected adults in Pakistan [16, 23]. Self-Help Plus (SH+), delivered in a large group setting accompanied by a self-help book was effective in reducing psychological distress in South Sudanese female refugees in Uganda [29].

The WHO has developed a group intervention for young adolescents (10 to 14-year-olds), exhibiting internalising problems (e.g., symptoms of depression, anxiety or distress) [10] called Early Adolescent Skills for Emotions (EASE). A key component of the EASE intervention is the involvement of caregivers in providing support to adolescents. EASE aims to increase the potential for scalability through: (1) a brief number of sessions; (2) group-delivered format; (3) combining evidence-based intervention components to target common symptoms of distress rather than focusing on a single mental health condition (i.e., transdiagnostic approach); and (4) designed to be implemented by trained and supervised lay providers (i.e., through ‘task sharing’). EASE is also being adapted and tested in Jordan, Lebanon and Pakistan.

Before evaluating interventions in new socio-cultural settings, it is essential to ensure that interventions are consistent with local needs and that intervention content is adapted for the socio-cultural context [13]. The adaptation of an intervention to a given socio-cultural setting must be balanced with maintaining fidelity to the key therapeutic ingredients [3]. There is some evidence to suggest that cultural adaptations to psychological interventions can improve effectiveness compared to interventions which are not adapted in this way [25]. Our aim in this study was to adapt the EASE intervention for use with Burundian refugee adolescents and their caregivers in refugee camps in north-western Tanzania.

## Methods

### Setting and population

The study was conducted in the Mtendeli refugee camp in northwest Tanzania, which is largely inhabited by refugees from Burundi. Burundian refugees have sought safety in Tanzania and other countries following periods of cyclical violence and protracted civil strife since independence. Refugees in Tanzania have fled Burundi following renewed political violence in 2017.

There are three refugee camps in north-western Tanzania, with Burundians forming 75% of the overall population [31]. Nearly half of the refugees in these camps are below the age of 18 years [34]. Mtendeli refugee camp was selected because the implementing partner, International Rescue Committee (IRC), had ongoing education and mental health and psychosocial support (MHPSS) programming in this camp in which EASE could be integrated. While the adaptation process was being conducted, Mtendeli housed over 40,000 people, many of whom continued to live in emergency shelters [32]. Voluntary repatriation efforts remain contentious, sparking anxiety and fear amongst this displaced community [17].

Adolescents fleeing conflict in Burundi have been exposed to violence, risk separation from their families, and experience a breakdown in traditional community support mechanisms. They are at increased risk of child labour, trafficking, exploitation and forced recruitment by armed groups [32]. Gender-based violence remains a prominent protection risk in the camps, especially for adolescent girls. Further, an educational needs assessment during 2018 highlighted the disruption of education and limited educational opportunities at the camp, with only 56% of Burundian children enrolled in schools across the three camps [1].

### The EASE intervention

The version of EASE adapted for this study is comprised of seven, 90-min group sessions and includes evidence-based techniques: psychoeducation, stress management, behavioural activation, problem solving and relapse prevention. There are also three group sessions (120 min) for caregivers which aim to increase capacity to support a child in distress, which include the following discrete strategies: psychoeducation, active listening, quality time, praise and showing interest in their child, reducing harsh discipline, caregiver self-care (including stress management) and relapse prevention for their child. The involvement of caregivers is supported by the WHO mental health Gap Action Programme (mhGAP) intervention guidelines, which recommend caregiver skills training for emotional and behavioural disorders in children [41]. Additional materials were developed as part of the EASE package and include a storybook, and workbook with practice exercises for adolescents and posters and handouts for both adolescents and caregivers, to support session delivery.

### Adaptation process

We aimed to adapt the EASE intervention for use with Burundian refugee adolescents over four different phases (Table 1). Throughout these phases we kept notes on a spreadsheet, structured in accordance with the dimensions for adaptation suggested by Bernal and Rodriguez [2]. Informed assent from participants < 18 years, together with witnessed caregiver informed consent was obtained from all participants. All adults provided witnessed informed consent.

**Table 1:** Phases of adaptation of the EASE intervention for displaced Burundian adolescents in Tanzania

Adaptation phase	Aim	Methods	Main outputs
Phase 1: Desk review	To synthesize existing knowledge on mental health among Burundian conflict-affected populations	Academic and grey literature databases searched using selected keywords	Worksheet developed to summarise information from 24 articles on cultural concepts of mental health, available services and culturally appropriate measures used with Burundian communities
Phase 2: Rapid qualitative assessment	To understand mental health concerns, experiences of violence and coping strategies of young Burundian refugees and preferences for seeking support from their own and other key stakeholders’ perspectives	Free listing (*n* = 61) and key informant interviews (*n* = 24)	List of most salient mental health problems and experiences of violence, and a thematic framework with in-depth information on perceived gender and age differences, barriers and facilitators to support seeking and ways of coping
Phase 3: Group cognitive interviews	To check that the intervention materials were easily and accurately understandable, acceptable and relevant for adolescents and caregivers	Participants (*n* = 20) shown core intervention materials to assess understanding and acceptability of materials	Response sheets to check comprehension by participants against intended meaning and preparation of a list of problem areas that needed to be adapted
Phase 4: Adaptation workshop	To review data from earlier phases and propose recommendations for adaptation	Group read through of intervention sessions and supporting materials, review of data from earlier phases and consensus on proposed adaptations	Adaptations log with suggested changes and corresponding Bernal codes

#### Phase 1: desk review

##### Aim

The aim of this phase was to synthesize existing published research focused on mental health among Burundian conflict-affected populations, with relevance to local mental health concepts, help-seeking behaviour, and supportive practices.

##### Procedures

The desk review built on guidance for desk reviews as published in “Assessing Mental Health and Psychosocial Needs and Resources: Toolkit for Humanitarian Settings” [43], which has been used to conduct desk reviews in various humanitarian settings [15]. We searched through free and/or publicly available databases to retrieve articles from Google Scholar, PubMed, PsycInfo and the IRC database. (The IRC database is an internal repository of selected articles relevant to the humanitarian sectors the IRC works in, including mental health, psychosocial support and women and children’s protection). Relevant grey literature was retrieved from MHPSS.net, ReliefWeb, ALNAP and from the websites of key agencies present in the region and working with Burundian adolescents [such as Plan International, Save the Children, United Nations High Commissioner for Refugees (UNHCR) and the International Committee of the Red Cross (ICRC)]. We selected articles that reported on young people’s MHPSS concerns. Primary or secondary, qualitative or quantitative or mixed method articles from the last 15 years were selected using a combination of key words (Table 2).

**Table 2:** Search strategy for the desk review

(Mental health services OR Psycho* services OR Mental health treatment OR Psycho* treatment OR Mental health care OR Psycho* care OR Mental health intervention OR Psycho* intervention OR Mental health support OR Psycho* support OR Mental health program* OR Psycho* program*) AND (Depression OR Anxiety OR Posttraumatic stress OR PTSD OR Common health disorders OR Common mental health difficulties OR Common mental health problems) AND (Burundi OR East African OR Tanzania OR Mtendeli OR Nduta OR Nyarugusu OR Democratic Republic of Congo OR DRC OR Congo OR Kenya OR Uganda or Rwanda) AND (Adolescents OR Children OR Young people) AND (Refugee OR Asylum seeker)	

In addition, we consulted with seven humanitarian mental health and psychosocial support and protection experts in the region via email and in-person to ask for recommendations on resources for the desk review.

##### Analysis

Relevant information was synthesised by using a spreadsheet that contained the following headings: objectives; design and tools; sample; mental health conditions; providers; training and supervision provided; type of intervention; measures; results and implications; and study limitations.

#### Phase 2: rapid qualitative assessment (RQA)

##### Aim

The goal of the RQA was to understand the mental health concerns of young Burundian refugees and preferences for seeking support from the perspective of adolescents, caregivers, and other stakeholders. We were additionally interested to understand the experience of violence, the effects of MHPSS concerns on routine activities, and commonly used coping mechanisms to manage distress.

##### Participants and sampling

We employed a maximum diversity sampling approach to conduct 61 free listing and 24 key informant interviews, continuously assessing for saturation of incoming information. A total of 85 individuals participated in the RQA (Table 3). Girls (*n* = 25) and boys (*n* = 19) were aged between 10 and 14 years. Caregivers (*n* = 45) included parents, teachers, community leaders and health or protection workers. Snowball sampling was used to identify participants; additionally, we relied upon community interviewers’ knowledge to identify community leaders, health and protection workers and teachers with regular team discussions to assess geographical coverage throughout the camp.

**Table 3:**
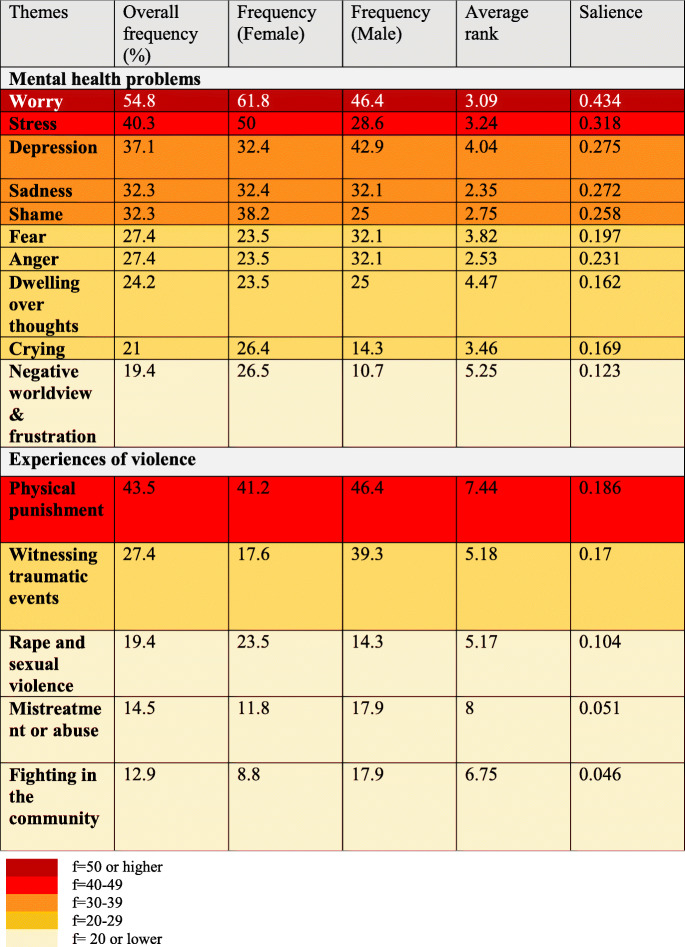
Main mental health problems and concerns related to violence

##### Instruments

The interview guides were based on the rapid ethnographic semi-structured interview tool 10 in the World Health Organization and the United Nations High Commissioner for Refugees (UNHCR) MHPSS needs assessment toolkit [43]. This tool is structured using tables to allow for easy data collection and analysis in low-resource humanitarian settings and was adapted for this study to break down questions by gender. Participants were initially invited to a free listing interview, and subsequently a sub-set of participants were invited to provide more detailed information during key informant interviews. The initial free listing interview asked participants to list the types of mental health problems and types of violence that may be experienced by Burundian adolescent girls and boys in this setting. This was followed by an in-depth exploration through detailed interviews of ways of coping, sources of support, changes due to displacement, perceptions of gendered patterns, information available on services and barriers to accessing these. We also explored preliminary acceptability of provider characteristics and perceived barriers and facilitators to accessing EASE. An additional consideration for the key informant interviews was a pre-determined focus of the qualitative enquiry on problems associated either with psychological distress (58% of the interviews) or with concerns related to violence (42%).

##### Procedures

All interviews took place at Mtendeli Camp between February and May 2018. Interviews were conducted in Kirundi with the support of incentive workers, that is, lay refugee workers who are paid a small monetary incentive according to employment regulations for refugee workers in Tanzania. Incentive workers were selected based on prior experience working with children and overall communication skills. All incentive workers received a two-week-long training on qualitative interviewing approaches and the study instruments. The training period extended to supervised interview practice over 2 weeks following the training. Where possible, interviewers were sex/gender-matched to participants. Interviews were conducted in pairs, with one person leading the interview and the second taking notes.

##### Analysis

The free listing interview format lends itself to rapid analysis based on handwritten notes, which were translated, typed and analysed for frequencies of response and relative position. All unique mental health problems and concerns associated with violence that were mentioned by participants were compiled and Smith’s Salience Index [27] was used to analyse the frequency and relative position of responses in the list provided.

Key informant interviews were transcribed verbatim, translated and typewritten for a thematic framework analysis [4], conducted using an Excel worksheet to describe brief summaries of each participant’s responses on all questions asked.

#### Phase 3: group cognitive interviewing

##### Aim

The purpose of the cognitive interviews was to check that the intervention materials were easily and accurately understandable, acceptable and relevant to the participants.

##### Participants and sampling

A Community Advisory Group (CAG) was formulated for regular consultations and community engagement. Through discussion with IRC providers, we selected adult, youth and adolescent participants for the CAG based on their availability to participate, good communication skills and an interest in community activities. Adult participants were parents and caregivers, teachers, health and protection workers and camp leaders. The purpose of the CAG was to seek rapid feedback on intervention materials during their initial development and adaptation [for example, to receive feedback on the appearance of the main character in the story book and select from sample options, or to understand where to hold qualitative enquires as part of the Rapid Qualitative Assessment (RQA), and so on]. The results of the adaptation process were shared with the CAG. The CAG also served the function of increasing participation, disseminating information, and enhancing community acceptability towards the intervention.

Four group cognitive interviews were conducted, one each with a group of adolescent girls, adolescent boys, mothers and fathers. Each group cognitive interview had five participants (i.e., a total of 20 participants), who were all recruited from the CAG, formulated at the onset of project activities and therefore, were all familiar with the overall goals of the programme.

##### Instruments

Intervention materials were purposefully selected based on discussions with the local team and other EASE implementing sites to select content which was hypothesised to potentially pose challenges for acceptability, relevance and comprehension in this context.

This included content which mentioned parental conflict, common coping strategies used by adolescents, adolescent emotions, suicide, and the EASE slow breathing exercise and EASE manging problems exercise. Cognitive interviews with caregivers were conducted similarly with the following components of the intervention selected for interview: talking about suicide with young people, alternatives to physical punishment, and caregiver self-care.

##### Procedures

An IRC Tanzania MHPSS Officer with a bachelor’s degree in psychology and previous work experience delivering psychological interventions with the Burundian refugee population, co-led the cognitive interviews in partnership with a Kirundi-speaking incentive worker from the community. All cognitive interviews took place at the IRC Wellness Centre in Mtendeli camp.

Adolescents and caregivers were shown selected core intervention materials in their translated Kirundi form and were asked for their reactions to it. They were asked to describe how they understood the selected excerpts of the storybook and related exercises in the workbook. Participants were also asked about how adolescents would relate to the situations, problems and feelings described in the in manual and materials, and how parents would understand the content relevant to the caregiver sessions. They were encouraged to describe any potential misunderstandings that may arise from the materials shown or components that may be less likely to be received well. For each problem area identified, suggestions were sought.

##### Analysis

Responses were noted on the cognitive interview response sheet and translations analysed qualitatively. Responses to the understanding of the materials were cross-checked against the intended meaning and themes of problem areas were identified for recommended adaptation. A list of community stakeholder recommendations for adaptation were prepared and reviewed during the adaptation workshop.

#### Phase 4: adaptation workshops

##### Aim

The goal of the workshop was to review data from earlier phases and propose recommendations for adaptation.

##### Participants

A two-part adaptation exercise was conducted, the first in Tanzania, and the second in Burundi. The first workshop part took place at the IRC field office in Kibondo, Tanzania, and was attended by six IRC staff members working on the EASE programme (including international, Tanzanian and Burundian community-based team members), a WHO consultant and a psychiatrist consultant from the Muhimbili University of Health and Allied Sciences (MUHAS) in Dar es Salaam, Tanzania. The second workshop was conducted in two sub-parts. The first part took place in Kigutu, Burundi, with seven mental health professionals from Village Health Works (VHW), which is a community-based primary healthcare facility delivering a mental health programme. The second part took place in Bujumbura, Burundi, with six IRC health and protection workers.

##### Procedures

The workshop comprised of: 1) a group read through of the EASE adolescent and caregiver sessions and supporting materials (the storybook, workbook, posters and handouts) in English involving all workshop attendees, and in Kirundi by those from the Burundian community; 2) followed by a review of the data collected in previous stages of the programme; and 3) concluded with ascertaining item-by-item recommendations for adaptation.

##### Analysis

Each recommended adaptation was discussed. Consensus was reached on selected recommendations.

We built on dimensions described as important when adapting psychological therapies as described by Bernal [3]. Building on these dimensions we developed a spreadsheet on which we systematically noted potential adaptations throughout all the different phases. A code corresponding to the different categories of the Bernal framework, that is, a) language, b) people, c) metaphors, d) content, e) concept, f) goals, g) methods, h) context, was assigned to each adaptation item and reviewed for consistency by all other members of the team.

## Results

### Phase 1: desk review

Twenty-four articles were identified in the desk review. Of these, seven employed primarily qualitative methods, eight used quantitative methods and the remaining nine employed mixed methods. Our main findings relevant to this paper were: (1) There is a small but growing body of literature on Burundian concepts and prioritised mental health problems [39, 37, 12] which are grounded within the socio-political environment; (2) there are validated tools to assess psychological distress in Burundian children and youth [8, 38, 18]; (3) while there is a gap of evidence regarding the effectiveness and efficacy of treatments, most interventions for young people in humanitarian settings, including with Burundian adolescents, have demonstrated positive outcomes [20, 36].

Within the broader framework of EASE, which was identified at the onset, adaptations were made to the intervention through this systematic, phased process, beginning with the desk review. Information from other psychological intervention studies with this population was helpful in learning what adaptations would be appropriate for culture and context so that the EASE intervention was most relevant to Burundian refugee adolescents living in refugee camps in Tanzania. Each phase of the adaptation process led to specific changes. For example, the desk review indicated that treatment approaches were closely linked with beliefs about causality, especially with suicide [37]. This led to adding text to the intervention manual to emphasise that talking about suicide will not cause suicide to happen and speaking openly with the child about difficult feelings when the caregiver is worried that there may be a risk could in fact help to prevent the risk of suicide. Religion as a coping strategy was assessed as ambiguous, being both helpful and unhelpful in the context of local conceptualisations of mental health problems. We decided to delete references to prayer in the intervention manual and included this as a training topic for discussion with facilitators.

### Phase 2: rapid qualitative assessment

Through free listing, we identified 95 mental health problems. After categorization, the most frequently mentioned mental health problems and concerns associated with violence are described in Table 3 along with Smith’s Salience Index.

#### Priority mental health concerns

‘Worry’ was reported to be the most salient mental health problem (salience index 0.434) amongst adolescent girls and boys, reported by nearly 55% of the respondents as amongst the top three problems experienced by early adolescents. Further, 40 and 37% of the participants reported that ‘stress’ and ‘sadness’ respectively were amongst the main five problems experienced by adolescents. The frequency of report of ‘stress’ was higher for women and girls at 50 compared with frequency of report by men and boys at 28.6, and the frequency of report of ‘depression’ was higher amongst men and boys (frequency = 42.9) compared with women and girls (frequency = 32.4). Depression has been understood variously across cultures and languages. In our exploration of what depression might look like in adolescents in this community, it was described by participants as consisting of (a) feeling low, (b) withdrawing from social interactions with peers, family and at school, (c) experiencing difficult thoughts. A third of the participants shared that ‘sadness’ and ‘shame’ were amongst the top three problems of Burundian refugee adolescents in Tanzania, with a higher number of females reporting this.

While a large proportion of the adolescent participants reported sadness as a top problem (44%), only 18% of the adult participants recognised this as a concern in need of priority attention, whereas anxiety-related difficulties were recognised more frequently by caregivers. Other relevant psychosocial distress complaints were ‘fear’, ‘anger’, ‘dwelling over thoughts’ with each problem reported by a quarter of the participants and frequency of report being similar across youth and adults.

#### Main problems associated with violence

The top problem associated with violence was ‘physical punishment’ for both boys and girls, reported by 43.5% of the respondents. ‘Witnessing traumatic events’ as a top problem was highlighted by over a quarter of the participants with a significantly higher number of reports by boys and men (frequency = 39.3) as compared with girls and women (frequency = 17.6); however, narratives of sexual violence as traumatic experiences for young adolescents were common and amongst the top five problems faced by girls (frequency = 23.5) in this community.

#### Impact on daily activities

To better contextualise findings regarding mental health of Burundian refugee adolescents, we aimed to learn more about how girls and boys spent their time in Mtendeli refugee camp, and which activities were most affected when they experienced mental health difficulties. Girls and boys reported their main activities as water collection, carrying firewood, cleaning, washing, cooking, praying, playing and studying. Water collection and carrying firewood were associated with risks of sexual violence for girls and boys. In addition, while girls were involved in household farming activities and were responsible for caring for their younger siblings, boys worked in brick-making and were expected to engage in household income generating activities. For boys, this implied leaving the camp for work (which may potentially expose them to a range of psychosocial and protection risks, including violence).

An ‘intelligent’, ‘polite’ and ‘obedient’ adolescent was described as a girl/boy doing well, and being good at studies, doing daily chores (e.g., cleaning), and being an effective communicator were important indicators of this. Caregivers’ responses indicated that they assessed boys’ well-being through outward indicators, such as playing, eating and smiling, but found it more difficult to identify characteristics of girls who were doing well. Therefore, it can be gathered that parents found it more challenging to identify signs of emotional problems and well-being of adolescent girls, who typically had fewer opportunities to play and interact with others.

#### Ways of coping

Nearly three fourths of the participants described two main ways of coping: a) praying and other religious practices to deal with stressors of camp life, and b) seeking support from camp-based health and protection services provided by humanitarian organisations. Over two thirds of the adolescents reported seeking advice from elders in the community, demonstrating the key role of respected community members in providing support. Additionally, over 40% described their preferred way of coping with psychosocial difficulties as working towards meeting their basic needs (e.g., food and school materials such as books, pencils, bag etc.), and 40% also described spending time with friends and family as a way of coping. Other coping strategies were thinking of a better future, being encouraging of each other, going to school, forgiving others, staying away from ‘bad’ company, and being patient. A potentially unhelpful or maladaptive coping strategy which was commonly reported was ‘not talking about one’s problems’.

#### Preferences

A preliminary exploration was conducted on the preferences for provider characteristics and delivery of the intervention. In summary, preferences were for the provider to take on a training and advisory function and be able to collaborate with community leaders. No preferences for age and gender of EASE facilitators were found. It was important that they were perceived as trustworthy by the community and that sessions be held at a convenient and safe place that was easy to access. Examples are discussed below.

First, the provider was seen as a trainer for adolescents and caregivers. For example, a mother shared, ‘*The facilitator would be someone who trains parents on how they can help their children with their problems, and also trains children on how to behave during a time of difficulties*’. It was important for parents that the intervention involved teaching adolescents the value of obedience and respect towards elders and community leaders, which was congruent with the characteristics of adolescents doing well. In a community affected by political and community violence, hatred was a common theme and both young people and caregivers suggested that improved social relationships were a desirable outcome of a psychosocial intervention.

Preferred provider characteristics included ‘being merciful’, ‘loving towards children’ and being ‘trusted by everyone’. Familiarity with community members and engagement with local leaders (e.g., block leaders, zone leaders, religious leaders) whose role was to ‘*advise, instruct, and assist’* was reported to be crucial by caregivers. Religious leaders suggested that the team of facilitators include people from diverse backgrounds, such as those with teaching experience, those with experience in child protection and other community members with leadership experience.

As mental health services for young people were limited at the camps at the time of this study, when asked about where adolescents would like to receive support, there was a diversity of responses based either on where child protection services were provided or where services for adults experiencing distress were available. A majority suggested that sessions be held at the IRC Wellness Centre, which provides psychological support for adults; others suggested that sessions be at child friendly spaces or schools or in the community. With regard to the timing of sessions, there was a preference for weekend sessions for school-going adolescents and caregivers.

### Phase 3: group cognitive interviews

Adaptations arising from the cognitive interviews were associated with (a) visual appearance of characters in the storybook and presentation of the camp context, (b) understanding of problem management activities for adolescents described in the EASE intervention materials, (c) cultural acceptance of content from the caregiver sessions.

Group cognitive interviews indicated that boys found the main character easier to identify with as compared to girls. It was advised that the name of the main character be changed, and the character strengthened to look Burundian. Recommendations were sought from Burundian incentive workers and the CAG on character revisions for adaptation (for example, vaccination marks, hairstyles and clothing in the storybook).

Cognitive interviews demonstrated that adolescents were able to understand Kian’s (renamed Niyo- a gender neutral and shortened Burundian name) story easily and found family and peer relationships relatable as described in the intervention materials. We also assessed comprehension of potentially complex exercises (such as the ‘feelings pot’ for emotion identification) and found that while these were novel activities, adolescents were interested in learning their use, however, needed more support. The problem-solving strategies were considered more complex, and an important recommendation for adaptation was simplifying these to ensure understanding. The EASE slow breathing activity was comprehensible. Finally, adolescents had difficulty relating to bird spotting as a leisure activity and this narrative was often misunderstood as bird hunting instead. For example, a young boy shared, ‘*What he (Kian) does is not what we do; he goes to hunt in the bush, but we go to school*’. This implied that there was a need for such activities to be more relatable in the cultural reality of the camp setting.

From the group cognitive interviews with caregivers, it was clear that caregiver sessions needed cultural adaptation to enhance acceptability of content. There were strongly held views about suicide in the community (for example, regarding supernatural powers causing suicide), which required additional training of facilitators and data collectors both to provide education about suicide and to increase knowledge of how facilitators could support participants in understanding suicide and its prevention. The use of physical punishment for disciplining was common (also reported as a top concern in the previous phase) and it was suggested that the session for caregivers on alternatives to physical punishment include specific examples of culturally acceptable alternatives (for e.g., prevention and monitoring strategies currently used by Burundian parents, such as keeping children busy, staying near and timing play).

### Phase 4: adaptation workshops

All suggested changes were logged using the Bernal dimensions. The most significant adaptations that were required included providing options for low literacy of participants, safety planning to address the high prevalence of sexual violence, simplification of strategies for the benefit of the end users and of lay facilitators, and implementation changes to consider involvement of refugee incentive workers. While the adaptations to the goals and concepts of the intervention were few, there were extensive adaptations across all other dimensions of the framework (that is, language, people, metaphors, content, methods and context). A summary of the main adaptations is presented in Table 4.

**Table 4:** Summary of adaptations

Bernal code	Adaptation made
**1.Language**	1. The phrase ‘caregivers and parents’ was used throughout the intervention due to cultural implications of the word ‘caregivers’ in Kirundi, which typically indicates foster parents only. 2. The word *ibishobo* literally translates to emotion but often implies a happy or exciting state of mind, so this was replaced by *inyiyumvo*, which was perceived to be understood as more inclusive of a range of emotions. 3. ‘Bullying’ was replaced with ‘beating’ or ‘provoking’. 4. The word ‘imagine’ was changed to ‘think about’ (e.g., think about sweeping the floor). 5. The term ‘emotional problems’ can be stigmatising; it was changed to ‘difficult feelings’. 6. Language was included to describe local idioms which were included in the translation, for example, *akabonge*, which connotes dwelling over what is lost along with social withdrawal and sadness. 7. Some somatic sensations related to emotions such as a ‘buzzing sensation’ or ‘face feeling hot’ were not easily identified, these terms were replaced with other bodily sensations or visible behaviours, such as ‘teeth showing’ for happy or excited feelings. 8. Self-harm was not easily understood, so we altered the language used to describe that this includes ending your own life as well. 9. Tentative statements were removed because ‘maybe’ was often perceived to mean ‘no’. 10. The concept of respect (in group sessions) can be understood differently in different contexts, therefore the word ‘non-judgemental’ was added to clarify the intended meaning.
**2. People**	1. The concept of the therapeutic alliance as relevant to working with children was added to the training materials in greater detail. 2. It was decided that the intervention would be provided in the community during the piloting stage and not at schools. This was because there was a large population of non-school going adolescents at the camp. In addition, we identified increased risks of sexual violence at school. 3. The main character was described and illustrated in as gender neutral a manner as possible. A gender-neutral Burundian name was chosen, *Niyo*, which can be used as a nickname for boys or girls. The names of caregivers were also adapted so they were easier to be identified as from the same community. Further, identifying information to make her/him look like children in this setting was added, such as a vaccination mark on the arm. We included instructions to the facilitators so that children could be asked to colour in so that the main character was easier to identify with, if needed. 4. It was decided that sessions would be held separately for boys and girls and ages 10–12-year olds and 13–14-year olds. Caregiver sessions would also be conducted in a gender segregated manner as this is more culturally appropriate and would encourage women and men to speak openly. 5. Adaptation to make activities appropriate to the age and gender of the group were made throughout, for example, that facilitators should choose age-appropriate session opening activities and provided examples (such as a game or a dance).
**3. Metaphors**	1. On an emotion recognition activity (‘feelings’ pot’) involving colouring, colour connotations may be different for Burundian children. It was highlighted that it is important that children use the colours in the storybook as an example only and alternative codes were suggested e.g. the use of shapes or drawings to illustrate an emotion. 2. Using positive ‘strength’ words was perceived as important and the qualitative enquiries demonstrated that positive qualities such as being caring, loving, patient, merciful and wise are prioritised. We added in the culturally appropriate metaphor to a key breathing exercise such that it was described as ‘breathing in strengths and saying the word ‘peace’ when breathing out. 3. Self-control was highlighted by community members, with a metaphor on ‘being the master of oneself’ being central. Caregivers as role models learning to manage their own feelings was added, emphasising its importance for strengthening the parent-child relationship. 4. ‘Running away’ was used as an example as part of the vicious cycle to explain how emotions can affect behaviours.
**4. Content**	1. Activities relevant to Burundian refugee adolescents were referenced throughout, Certain other common activities such as fetching water and collecting firewood, were purposefully not referenced as both of these were associated with increased risk of sexual violence. Facilitators were then advised on how to manage these if these examples came up. 2. References to prayer as a potential coping strategy were removed due to the association between some religious settings and violent practices in the context of mental health and suicide. 3. A community awareness strategy was developed to increase the acceptability of the intervention by the community. 4. Symptoms of shame in describing vicious cycles were added. 5. Losing parents or being orphaned was included as a stressor for adolescents and in discussions on self-care with caregivers. 6. Child Friendly Spaces as an example location where behavioural activation activities could be conducted was added. 7. The addition of seeking social support from ‘a trusted adult’ was given in order to account for some children not having parents and some children not feeling like a teacher is a safe adult for them. 8. Further information was added for caregivers on the impact of parental stress on well-being of adolescents and examples referencing stressors experienced by caregivers at the camp. 9. What it means to stay well in this community was included such as spending time with friends and family, praying, seeking advice from others, being patient etc. 10. Bird watching was challenging to identify with for young adolescents and likely to be misunderstood as bird hunting, so a caveat was introduced to indicate that alternative examples that were locally could be used instead. 11. Missing school was taken out as an example from the manual as it was associated with parents preventing children from going to school, which had a different mean from what was intended. 12. In the training manual it is added that you cannot change behaviours and social norms overnight. 13. Revenge as an unhelpful way to solve problems was highlighted in training as it is a commonly used strategy. 14. Examples were added of quality time spent with children, such as eating together, going for a walk together on the weekend, sitting under a tree, telling simple stories, joining parents during farming activities, and asking your child ‘how are you?’ or ‘how was your day?’.
**5. Concepts**	1. Content on myths regarding causality, especially as they relate to suicide, were included. 2. The session ‘managing my problems’ was revised to provide a simpler explanation without losing core instructions. This was especially important for younger adolescents. 3. It was emphasised that somatic complaints can be related to distress, which may need to be followed up by a health worker.
**6. Goals**	1. Safety planning strategies were emphasised in the training and in the facilitators’ version of the manual due to the risk of sexual violence.
**7. Methods**	1. While discussing alternatives to physical punishment with caregivers and adolescents, a more tailored explanation was provided, for example, by saying hitting instead of shouting. Further, emotional abuse was added in addition physical punishments. 2. Illiteracy options were introduced throughout the intervention manual and facilitators trained in engaging with children who could not read or write. This was of particular relevance to the ‘managing my problems’ session. This also applied to caregiver sessions as literacy was not high. 3. Training for facilitators included ways of managing disclosure of experiences of sexual violence as this was prevalent in the community. 4. Children may be reluctant to share feelings, so facilitators were trained and advised that this is a common experience in children’s interventions. 5. Guidance was added to the training manual on how to manage time when managing distress and strong emotions during the session (e.g., crying). 6. In case of children missing the intervention sessions, additional instructions were included in the review of the previous session, in order to allow participants to catch-up with missed content. 7. The vicious cycle described in the intervention was complex; instructions were added on how facilitators could explain it step-by-step. 8. Concepts of planning and breaking things down in written form were not familiar adolescents. Additional training was provided on these concepts.
**8. Context**	1. References to the camp context were included and those not relevant excluded. For example, removing references to phone usage/reminders as caregivers almost never had phones. 2. In a breathing exercise described in the storybook, adolescents were advised to keep a balloon under their pillow, however, refugee adolescents in this setting do not sleep on pillows. It was agreed that instead of a balloon under their pillow, the picture indicate a ball of clothes used as a pillow, which was more common, and that, as an example, the balloon be kept besides where one sleeps and not under the pillow. 3. Training for all staff on suicide protocols was expanded to increase facilitators’ confidence and comfort in talking about suicide, as there was a large stigma associated with suicide in the community. 4. It was highlighted that all efforts should be made to ensure confidentiality if sessions were held in outdoor spaces. Consistency in location was to be maintained throughout for piloting to asses any effects on intervention delivery. 5. There was a clear preference for weekend sessions by adolescents and caregivers/ We reached consensus that the feasibility of implementing this would be assessed in the next phase. 6. Facilitators and supervisors were aware that references to child labour were to be managed by the supervisors in accordance to child protection procedures. 7. Childcare facilities for caregivers attending EASE sessions were not feasible at this stage, however this may be a barrier for mothers, so facilitators encouraged them to bring another family member along to care for the young child. 8. The storybook ends with the family moving to a new town but this was not appropriate for refugees, so the end was altered to reflect a similarly themed but context appropriate story, for example, with the father leaving the camp to earn money. 9. Exercises were adapted so that they could be done within the constraints of a refugee camp and facilitators were guided to adapt EASE exercises to the camp context where helpful. 10. References to food and a good diet were problematic, these were removed where possible as discussions may be derailed and cause tension. Facilitators were trained to use this as an example minimally and to manage discussion if derailed.

## Discussion

This study sought to inform changes to a brief, transdiagnostic, group psychological intervention in a humanitarian context through a systematic cultural adaptation process. Research was conducted as part of the formative work in preparation for piloting the EASE intervention with Burundian adolescents and their caregivers living in refugee camps in Tanzania. Systematic methods of cultural adaptation of psychological interventions have been documented for some time, such as the method of cognitive interviewing [35], however there remains a lack of culturally adapted evidence-based treatment programmes for adolescents in low-resource settings. This contrasts to the literature indicating that culturally and contextually modified interventions significantly enhance effectiveness [28]. It is anticipated that this approach will maximise effectiveness, relevance and acceptability by tailoring to culture and context. A comprehensive approach across various dimensions of the intervention (e.g., language, concepts, content etc.) allowed for adaptations at multiple levels of the complex and heterogenous construct of culture [14]. Further, it is hoped that the clear systematic documentation through the different phases of the study (using the Bernal framework) provides valuable information on the cultural utility of the adapted intervention [7].

Adaptations were largely made across four main areas: (1) to contextualise to the camp setting and for adolescents and caregivers from Burundian socio-cultural settings, (2) to address the prevalence of sexual violence, (3) to adapt for low literacy, (4) to simplify strategies for problem management. These are discussed below.

This intervention, focused on internalising symptoms and supporting caregivers in responding to the adolescents’ distress, was deemed likely to be responsive to the needs expressed in the qualitative enquiry. In particular, some internalising problems (for example, sadness) were not commonly recognised by caregivers (but were by adolescents) whereas externalising problems (such as anger) were similarly reported by adolescents and caregivers. We also included in the intervention materials language commonly used locally to describe adolescent distress in the context, such as shame and embarrassment. Findings from the rapid qualitative assessment also consolidated that it was important to address young people’s experiences with violence as routine activities, such as water or firewood collection, were associated with risks of sexual violence. There were also reports of corporal punishment in school settings. Resultantly, one of the ways of managing this problem was by avoidance, perceived by parents as an avoidance of responsibilities and, in turn, reported to be a cause of physical punishment.

Participants highlighted the need for an independent facilitator to ensure that while the programme focussed on adolescents it should also build capacity of caregivers to provide the necessary support with the challenges faced by young people in this community. Adaptation also included contextualising caregiver sessions for Burundian refugee parents, for example, by discussing respect, highlighted as important in the community, but emphasising mutual respect and reciprocity. We also gathered that parents found it more challenging to identify signs of emotional problems and well-being of adolescent girls, who typically had fewer opportunities to play and interact with others, that is, fewer outwards responses that were typically identified by parents. Further research on gender differences between girls’ and boys’ responses to distress in this community is useful in future adaptations of psychosocial interventions.

Importantly, these contextual risks highlighted the need to build in structured safety planning within the programme. This required specific training on how to identify participants who may have experienced violence and how to manage disclosures of violence, by providing training to facilitators on how to ask sensitive questions and providing basic counselling skills to facilitators. This also required that facilitators understand their role and responsibilities when sexual violence was disclosed, for example, contacting their supervisor and providing a referral for additional support. Guidance was included in the intervention manual for specific sessions where young people might be more likely to disclose experiencing abuse. Guidance was also provided in behavioural activation and problem-solving strategies to ensure that activities which adolescents elected to engage in would not increase their risk.

Cognitive interviews highlighted the community’s need for recognition of the unique circumstances that Burundian refugees encountered in camps in Tanzania. Contextual modification also included adaptations to account for low literacy, for example, providing drawing-based activities rather than writing. Facilitators were trained in how to support participants with low literacy, for example by checking their understanding of content verbally, or by not requiring written notes as part of the in-session and take-away exercises. Likewise, the training and intervention materials for facilitators were revised to enhance their comprehensibility and thus strengthen anticipated fidelity to implementation.

An important adaptation involved simplifying existing strategies for problem solving and behavioural activation. The process of breaking down a problem into smaller or graded parts (a component of both these strategies) was hard to understand for young people and complex for facilitators. This was simplified by revised instructions and enhancing the training for facilitators on how to explain the vicious cycle (related to behavioural activation) as well as the steps involved in breaking down tasks (for both components).

Adapting an intervention for suitability to camp context posed some challenges, such as limitations on the extent of activities that could be considered for behavioural activation in the context of safety and resource issues. For example, we explored a range of other play-based interests of children with three criteria for selection of examples: (1) the activity should be free, that is, there should not be any cost implications and associated inequity for the family, (2) the activity should be gender neutral (3) the activity should be such that it could be done alone or with others (e.g., with a group of friends), (4) the activity should not be associated with any known or potential risks (e.g., firewood collection). We found that the range of activities available to children meeting these criteria were limited, however suggestions from incentive workers included using an empty rice sack to play a game that involved jumping.

Task-sharing approaches in global mental health have prioritised primary health care providers and community workers [21] and while our approach has been aimed at achieving maximum partnership with the community [6] by employing incentive workers with diverse backgrounds (as advised by participants during the RQA), we encountered challenges around capacity and retention of short-term staff, and national restrictions on refugees in paid employment. Furthermore, consistent communication with incentive staff was challenging because mobile phones could not be provided to incentive staff and limited in-camp transportation decreased the geographical reach.

### Limitations

We note several limitations of this study. (1) Only literature in the English language was reviewed as part of the desk review despite English not being the first or second language for most Burundian communities. The review would have benefited from inclusion of academic and/or grey literature in Kirundi, Swahili and French. (2) Rapid ethnographic methods imply that long-term participant observation was not possible, which may have an impact on the depth of information. (3) It was difficult to build probing skills of refugee incentive workers, who may question normalised cultural experiences less than external social scientists. (4) Translation remained challenging as there were many dialects of Kirundi spoken by the refugee community.

## Conclusions

A multi-phased approach to cultural adaptation of a psychological intervention allowed for diverse data sources and multiple stakeholders’ involvement in informing participatory intervention development. Adaptations consisted of both minor changes (e.g., changes to language) and more substantial changes (e.g., prevalence of sexual violence). A majority of changes were across dimensions of language, people, metaphors, content, methods and context and changes to goals and concepts were smaller, which aligns with findings from a systematic review of culturally adapted mental health interventions [9]. Adaptations were additionally retained for the generic EASE manual, namely, to include options for low literacy, and simplification of facilitator instructions for exercises and concepts We also note that system-level contextual challenges around legislation relevant to refugees’ involvement in service provision (e.g., paid employment) and their impact on research implementation processes can be challenging to overcome, however, a comprehensive approach to cultural adaptation of a psychological intervention is feasible in low-resource humanitarian settings. We anticipate that the adapted EASE intervention will be acceptable, understandable and relevant for adolescents and caregivers and is likely to be well suited to address the needs of Burundian refugees living in camp settings in Tanzania. Culturally adapted EASE may also be useful to other refugee communities and the intervention will continue to be informed by research from other EASE sites (Jordan, Pakistan and Lebanon), and by further piloting with Burundian youth in Tanzania (manuscript submitted).

## Supplementary Information

**Additional file 1: Table S5.** A sample of the adaptations log

## Data Availability

The datasets generated and analysed during the current study are available from the corresponding author on reasonable request.

## Abbreviations

CAG: Community Advisory Group
EASE: Early Adolescent Skills for Emotions
IRC: International Rescue Committee
JHU: Johns Hopkins University
KII: Key Informant Interview
LMIC: Low- and Middle-Income Countries
mhGAP: Mental health Gap Action Program
MHPSS: Mental Health and Psychosocial Support
MUHAS: Muhimbili University of Health and Allied Sciences
PTSD: Post Traumatic Stress Disorder
RQA: Rapid Qualitative Assessment
UNHCR: United Nations High Commission for Refugees
VHW: Village Health Works
WHO: World Health Organization
