# Supplementary material for: Cultural adaptation of a scalable psychological intervention for Burundian refugee adolescents in Tanzania: a qualitative study
Source: Confl Health. 2021 Sep 27;15:73. doi: 10.1186/s13031-021-00391-4 (PMC8477522; doi:10.1186/s13031-021-00391-4)
Supplement: Supplementary file 1 — Additional file 1: Table S5. A sample of the adaptations log [file 13031_2021_391_MOESM1_ESM.docx]

**Cultural adaptation of a scalable psychological intervention for Burundian refugee adolescents in Tanzania: a qualitative study** **– Supplementary material**

*Table 5: A sample of the adaptations log*

| **Issue** | **Recommendation** | **Source** | **Impact** | **Change** | **Code** |
| --- | --- | --- | --- | --- | --- |
| Differences in the way literate and non-literate adolescents are engaged | Make changes to the intervention manual in the introduction chapter and throughout activities for illiteracy options, and include section in training manual on adjusting style for literate or illiterate participants | Read through | Generic,  Adaptation  & Training | Yes | Methods |
| Implication of the word caregiver is non-biological parent in its Kirundi translation | Use ‘caregiver and parent’ throughout the intervention materials | Read through | Adaptation | Yes | Language |
| Sexual violence is amongst the top problems faced by adolescent girls | Include safety planning in problem management strategies and ensure facilitators are aware of referral protocols | RQA | Adaptation, Training & Implementation | Yes | Content |
